# Hereditary colorectal, gastric, and pancreatic cancer: comprehensive review

**DOI:** 10.1093/bjsopen/zrad023

**Published:** 2023-05-10

**Authors:** Toni T Seppälä, Richard A Burkhart, Bryson W Katona

**Affiliations:** Faculty of Medicine and Health Technology, Tampere University, Tampere, Finland; Department of Gastrointestinal Surgery, Tampere University Hospital and TAYS Cancer Centre, Tampere, Finland; Applied Tumor Genomics Research Program, University of Helsinki, Helsinki, Finland; Department of Abdominal Surgery, Helsinki University Central Hospital, Helsinki, Finland; Department of Surgical Oncology, Johns Hopkins University, Baltimore, Maryland, USA; Department of Oncology, Sidney Kimmel Comprehensive Cancer Center, Baltimore, Maryland, USA; Division of Gastroenterology and Hepatology, Department of Medicine, Perelman School of Medicine, University of Pennsylvania, Philadelphia, Pennsylvania, USA

## Abstract

**Background:**

Inheritance patterns show familial clustering of gastrointestinal cancers, and multiple germline conditions have now been identified that predispose to colorectal, gastric, and pancreatic cancers.

**Methods:**

A narrative review based on recent relevant literature was conducted.

**Results:**

Lynch syndrome, formerly known as hereditary non-polyposis colorectal cancer, increases the risk of several abdominal cancers, with the highest population prevalence. Familial adenomatous polyposis and some of the more infrequent polyposis syndromes have distinct characteristics affecting various organ-specific cancer risks. Hereditary gastric and pancreatic cancer syndromes include those also causing colorectal cancer, while additional genetic disorders predisposing only to upper gastrointestinal malignancies have been recognized more recently. Diagnosing and managing hereditary cancer syndromes requires multidisciplinary expertise and may be best managed in tertiary centres, with a need to consider patient preference and ensure shared decision-making.

**Conclusion:**

Several germline conditions predispose to colorectal, gastric, and pancreatic cancer, which inform identification, surveillance regimens, prevention, cascade screening, counselling, and surgical management. The authors describe developments in the hereditary origin of colorectal, gastric, and pancreatic cancer with current recommendations in surveillance and surgical management.

## Teaser

Hereditary gastrointestinal cancers are under-diagnosed and require attention for identification and differential diagnostics with easy access to germline genetic evaluation to confirm the diagnosis. Awareness of multiple predisposing conditions to cancers in various organs, also outside the gastrointestinal tract, is imperative in identification and prompting surveillance and management according to condition-specific clinical guidelines available.

## Introduction

Hereditary cancer refers to an increased probability of developing a malignant tumour due to an inherited genetic defect, polygenic risk, epigenetic factor, or otherwise unknown trait. Germline genetic predispositions can increase the risk of multiple different cancer types in various organs, often at relative early onset, causing a syndromic phenotype with distinct features. Sometimes a genotype–phenotype correlation is less evident, and only systematic tumour and germline testing may identify a hereditary condition. Hereditary cancers caused by germline defects often result in particular molecular alterations that open opportunities for precision medicine by targeted therapies^1^. Effective cascade testing of relatives should be used as an adjunct to systematic tumour and genetic testing and may improve outcome through surveillance and preventive measures^2^.

If the risk-modifying factor in a phenotypically high-risk family remains unidentified, the high familial risk may warrant increased surveillance and continued research to identify the responsible genetic factor. Clinical phenotypes have been used to create diagnostic criteria, but the studies the criteria are based on have often been influenced by ascertainment bias, that is returning the inclusion criteria as results of the work.

The most well-known gastrointestinal manifestations of inherited cancer syndromes relate to a high risk of colorectal cancer (CRC), such as Lynch syndrome (LS) and familial adenomatous polyposis (FAP). Both of these are associated with an increased risk of extra-colorectal malignancies, such as biliary tract and pancreatic in LS^3^, and gastric and small bowel cancers in both^3–5^. Hereditary diffuse gastric cancer (caused by for example *CDH1*) and hereditary pancreatic cancer (caused by for example *BRCA1/2*) have been defined more recently based on identification of gene defects in high-risk phenotype families. The availability and uptake of whole genome sequencing and broad genotyping panels has resulted in the identification of novel genetic associations (for example common low-penetrance alleles that confer a small increased risk), for which surveillance and management guidelines have not yet been developed.

This narrative review aims to summarize the latest guidance on identification, surveillance, and management of the most clinically relevant hereditary cancer syndromes relating to gastrointestinal cancer. Whilst some gene defects may result in cancers at multiple sites, the current review is structured by site for clarity and limited to syndromes predisposing to CRC, gastric cancer, or pancreatic cancer.

## Hereditary colorectal cancer

About 20–30 per cent of CRCs have a hereditary component^6^. Hereditary CRC risk refers to previously diagnosed cancers in first-degree relatives (FDRs) of the proband in question. Where one or more FDRs are affected, the proband has a family history of CRC categorized as average, moderate, or high based on the number of FDRs affected and age of onset^7^. High familial risk is likely to represent a known or as yet unidentified genetic predisposition to risk of cancer, and may be managed according to respective gene-specific guidelines. In large studies based on systematic next-generation sequencing (NGS) germline testing for cancer susceptibility variants, germline pathogenic variants have been diagnosed in over 7 per cent of all CRCs^8,9^.

### Family history of colorectal cancer

High CRC risk is assigned to probands with a cluster of at least three FDRs with CRC at any age in at least two generations, fulfilling the Amsterdam criteria^10^ for a high risk of a hereditary condition, but in which no underlying germline variant has been identified. The moderate-risk category includes probands with one FDR diagnosed at early onset, that is before 50 years of age, or two FDRs in the same kinship at any given age. Probands with no family history, or family history not fulfilling criteria for moderate or high risk are considered to be at average risk^7^. Whilst the presence of adenomas in FDRs does not generally indicate an increased risk in the proband, significant polyp load may indicate a multiple colorectal adenoma (MCRA) phenotype (10 or more adenomas but no constitutional variant detected) and stimulate increased surveillance.

The lifetime incidence of CRC is increased two- to six-fold in moderate- or high-risk probands compared with the general population^11^. Testing for deficient mismatch repair (dMMR) or microsatellite instability (MSI) in the affected family member’s previously resected tumours should be pursued to identify possible LS or other predisposing syndromes. Probands in the moderate- or high-risk category or those with an already identified familial or personal history of dMMR or polyposis should be referred to a specialist service for surveillance. Average-risk individuals may be managed in primary care with screening programmes targeting the general population. Early-onset CRC (eoCRC) denotes those diagnosed with a CRC before 50 years of age, and distinct guidelines exist for their risk and therapeutic management outside the scope of hereditary conditions caused by pathogenic germline variants that are found in about 13 per cent of those with eoCRC^12,13^.

**Fig. 1:**
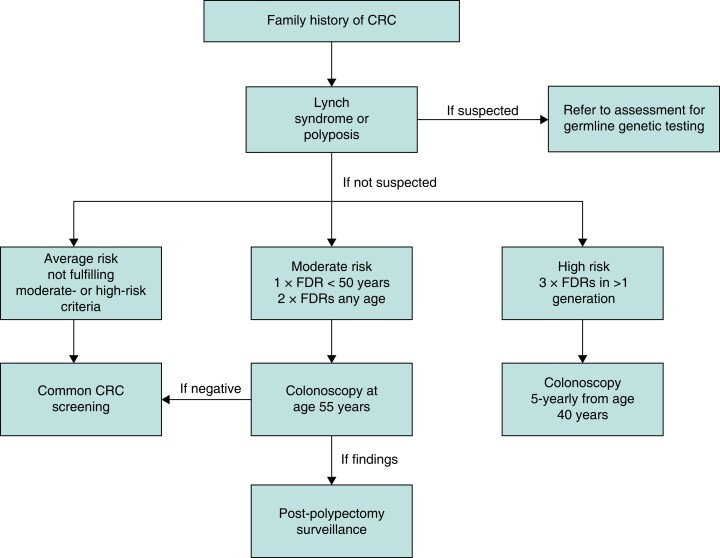
Surveillance recommendations for individuals with a family history of CRC CRC, colorectal cancer; FDR, first-degree relative. Modified from British Society of Gastroenterology recommendations^7^.

Probands with a moderate familial CRC risk should have a one-off colonoscopy at 55 years of age, while those with a high risk should undergo a 5-yearly colonoscopy from 40 to 75 years of age^7^, or at an age 5–10 years before the earliest CRC case in the family^14–16^. A screening and surveillance guidance for familial CRC risk is outlined in *Fig. 1*, adapted from British Society of Gastroenterology (BSG) guidance^7^.

### Lynch syndrome

LS is the most prevalent predisposing germline condition for hereditary CRC, and is caused by pathogenic germline variants in one of the mismatch repair (MMR) genes (*MLH1*, *MSH2*, *MSH6*, or *PMS2*) or epigenetic silencing of *MSH2*. It was first named as cancer family syndrome^17^, and later denoted as hereditary non-polyposis CRC (HNPCC), until named in 2003 after Dr Henry T. Lynch^18^.

#### Epidemiology

Pathogenic (PV) and likely pathogenic (LPV) MMR germline variant alleles are estimated to have a prevalence of up to 1 : 226 in the general population^19^, but estimates as high as 1 : 125 have been proposed^20^. An autosomal dominant pattern of inheritance has been demonstrated with the presence of one allele of PV/LPV adequate to cause the phenotype, which will itself vary by gene involved. Homozygous carriership or the presence of two separate germline MMR PV/LPVs results in the rare phenotype of constitutional mismatch repair deficiency (CMMRD), which predisposes to high childhood and adolescence incidence of various cancers with a dMMR phenotype. LS-associated heterozygous PV/LPVs are considered to result in 3–5 per cent of all CRC^21^.

#### Penetrance and expressivity

Most historical estimates of penetrance, that is the lifetime cancer risk, have been based on identifying a familial inheritance pattern. Such selection based on phenotype is at risk of ascertainment bias, leading to overestimation. In estimates based on historical segregation analysis, *MLH1* and *MSH2* are quoted to have a lifetime incidence of any cancer of up to 80–90 per cent without risk-reducing surveillance, whereas *MSH6* and *PMS2* have been linked with more moderate 40–60 per cent penetrance. Risk of CRC has been retrospectively estimated to be 40–50 per cent for *MLH1* and *MSH2*, and 10–20 per cent for *MSH6* and *PMS2*^22,23^.

Risk estimates from 2015 onwards have been largely based on prospective observational data generated by the Prospective Lynch Syndrome Database (PLSD)^24^, an international open initiative gathering registry data from specialized LS centres throughout the Western countries and Australia. Inclusion criteria for the PLSD have been a planned surveillance colonoscopy without cancer for individual PV/LPV carriers, which differs from the previous estimates of tracing families back in their cancer history, and therefore rather reflects the cancer risks under surveillance contact to healthcare than true penetrance. The PLSD quotes overall cancer incidence from 25 to 75 years of age up to 75–85 per cent for *MLH1* and *MSH2*, 42–62 per cent for *MSH6*, and 34 per cent for *PMS2* depending on gender^3,25^. These estimates are derived from 6350 LS carriers under surveillance for a total of 71 000 follow-up years, and can be seen at the interactive website www.plsd.eu. Of course, these figures may not represent the natural course of LS given interventions, namely polypectomy, which LS carriers might undergo as a result of surveillance colonoscopy. Lifetime cumulative incidence of CRC between 25 and 75 years of age is 48–57 per cent for *MLH1*, 47–51 per cent for *MSH2*, 18–20 per cent for *MSH6*, and 10 per cent for *PMS2* depending on gender^25^.

As a general notion to CRC occurrence, *MLH1* and *MSH2* are high-penetrance genes, *MSH6* is of moderate penetrance, and *PMS2* is a low-penetrance gene. The risk of cancer varies substantially based on gene and gender and so management should reflect this, rather than treating LS as one general entity^26^. Emerging evidence indicates that CRC risk varies between LS carriers with the same gene variant, suggesting modifiers of risk such as polygenic or epigenetic, immunological, or environmental factors^27^.

#### Carcinogenesis

At the molecular level of LS-associated dMMR cancer development, a constitutionally pathogenic, inherited, germline allele of an MMR gene, present in every cell, is followed by the loss of heterozygosity (LOH), that is loss of function of the second allele in the cell starting the cancer. The LOH results in a reduced capacity to produce a functional MMR protein, which impairs the function of the MMR complex. As the name suggests, MMR deficiency leads to the inability of the cell to correct nucleotide mismatches during DNA replication, which in turn causes accumulation of such mismatches throughout the cell’s genome^28^. This is observed as an increased abundance of mono-, bi- and multi-nucleotide repetitive sequences that occur randomly, but which may also frequently occur at non-random genomic positions, which can be detected as the genomic hallmark, microsatellites. Accumulation of microsatellite errors is denoted as MSI. Moreover, the mono- and di-nucleotide repeats cause a genomic frameshift that disrupts following sequences, which leads to a dysfunctional end product, that is a truncated protein^29^. MSI cells typically remain chromosomally stable, but possess a hypermutated phenotype that is prone to be captured by the immune response. However, when mutations occur within a tumour suppressor or oncogenes, carcinogenesis is initiated by accumulating necessary functional advantages to the developing cancer cell, facilitating immune escape and commencement of neoplasia formation when no longer eradicated or detained by the host immune response^30,31^.

CRC in general is considered to follow an adenoma-carcinoma sequence^32^, which may be modulated by screening and secondary prevention by removing precursor lesions (adenomas) via colonoscopy and polypectomy. In LS, efforts to prevent CRC by endoscopy have not been as successful, with incident cancers often diagnosed in-between regular surveillance colonoscopies^33,34^, a phenomenon that has resulted in significant research and hypotheses relating to carcinogenetic processes leading to dMMR CRC^30,35^.

It is unquestionable that dMMR CRC develops rapidly^36^, even between yearly or 3-yearly colonoscopies^33,37^. The adenoma-carcinoma sequence may be accelerated, and there is even debate that the precursor adenoma, which should be endoscopically detectable, may be skipped^30,38^. Nevertheless, normal-looking colorectal mucosa of random, previously healthy, MMR PV carriers has been demonstrated to contain multiple crypts that have lost their ability for MMR, and it is debated whether these dMMR crypt foci are precursors to manifest neoplasia^39^. In addition, the normal mucosa of healthy MMR PV/LPV carriers has been shown to contain an increased average abundance of CD3-, CD8-, and FOXP3-positive immune cells^40^ compared with LS cancer patients and proficient MMR cancer patients, and that time to CRC development is correlated to the relative abundance of these immune cells^41^.

Subsequent somatic events of cancer cells differ by the germline MMR gene background in developing CRCs. *MLH1*-deficient CRCs are more often *CTNNB1* (β-catenin) mutated and not *APC* mutated, whereas *MSH2*-associated CRCs almost always have *APC* mutations responsible for Wnt up-regulation^37^. In *PMS2*-deficient cancers and adenomas, it has been shown that the *KRAS* mutations take place earlier in the course of development than dMMR, which may indicate that MMR deficiency does not drive the carcinogenesis in *PMS2*-associated CRC^42–44^.

#### Clinical presentation

Pathogenic MMR germline variants predispose to an increased lifetime incidence of both gastrointestinal and extra-intestinal cancer. The cancer spectrum in LS includes at least increased risk of CRC, endometrial, ovarian, duodenal and small bowel, biliary tract, pancreatic, gastric, upper urothelial, bladder, prostate, skin, and brain cancers, as well as some sarcomas^25,45^. Some cancers are more associated with specific genes, such as urothelial, prostate, and brain cancers, which are closely linked to *MSH2* variants^3^. CRCs and endometrial cancers are detected in carriers of all four genes, yet with different ages of average onset.

The risk of CRC increases rapidly from 25 years of age onwards in *MLH1* and *MSH2*, causing early onset of CRC with a median age of 45–50 years. Onset of CRC is later for *MSH6*, with few or no cancers detected before 30–35 years of age, and the risk of CRC in *PMS2* PV carriers before 50 years of age is negligible if undergoing active colonoscopy surveillance^25,46^. Colonoscopy may reduce the CRC risk in *MSH6* and *PMS2* genotypes more than it does in *MLH1* and *MSH2* due to less frequent involvement of the dMMR crypt foci pathway. The risk of synchronous tumours in the colorectum but also in other organs is increased^47^, and identifying metachronous cancers by evaluating the entire colorectum and gynaecological organs before therapy or surgical management is important, and may affect decision-making.

The cumulative risk of adenomas and advanced adenomas in *MSH2* carriers is higher than in *MLH1* carriers under colonoscopy surveillance, whereas there is no difference in the cumulative incidence of CRC between the two^37^. LS-associated CRCs are more likely to appear first in the right hemicolon for reasons that remain unclear. They present more often with a mucinous component, dense immune infiltrates, and morphological intratumoral heterogeneity^28^.

#### Identification of Lynch syndrome

The Amsterdam criteria were developed to identify individuals and families in which to study genetic predisposition, and were later updated as Amsterdam II and Bethesda criteria^10,48^. While the criteria were never meant as a tool for ascertainment of individuals for clinical genetic testing, they have since been used for that purpose. Over two decades ago, universal tumour screening by MMR immunohistochemistry or MSI testing by PCR was recommended for all new CRCs^49^, and implemented into clinical guidelines^50^. Diagnosing dMMR/MSI has now gained direct therapeutic relevance with the use of immune checkpoint inhibitors (CPI) to treat metastatic dMMR/MSI solid cancers^51,52^. In cases where MSI or the loss of protein staining of MLH1, MSH2, MSH6 and/or PMS2 is detected, germline testing is warranted to confirm or rule out constitutional pathogenic variants in these genes underlying the molecular phenotype. If MLH1 staining is deficient, the presence of *BRAF* V600E in testing by immunohistochemistry (IHC) protein staining, PCR, or sequencing, or the hypermethylation of *MLH1* with pyrosequencing effectively rules out *MLH1* constitutional variants and therefore excludes LS without genetic germline testing^53^. Families fulfilling the Amsterdam criteria but with the phenotype containing proficient MMR CRCs have been denoted as familial CRC type X, with various candidate genes, and some of these cases have been proposed to harbour for example *RPS20* pathogenic germline variants.

IHC staining of MMR can be successfully performed from endoscopic preoperative biopsies without compromising the quality of the staining^54^. Meanwhile, novel germline testing pipelines may be able to return panel NGS results in 2–3 weeks, facilitating the diagnosis of LS in CRC before surgery. A preoperative LS diagnosis has implications for surgical decision-making; however, the diagnosis cannot be based solely on the MMR/MSI status, but requires a demonstrated pathogenic variant to confirm the diagnosis^55^.

#### Surgery

LS patients treated for CRC have a substantially high risk of metachronous CRC^56–59^. If diagnosed before surgery, *MLH1* and *MSH2* carriers with a colonic cancer should be considered for a subtotal colectomy with ileosigmoid or ileorectal anastomosis, to reduce the risk of metachronous colon cancer^55,60^. Extended surgery for *MSH6* and *PMS2* carriers is not currently recommended due to a lower risk of metachronous CRC^55^. Standard oncological pretherapy and rectal resection based on local advancement is recommended for rectal cancer as the first CRC in LS carriers, and total proctocolectomy is currently not recommended in this situation^55^. Stand-alone prophylactic procedures solely for risk, without existing neoplasia as an indication, for example prophylactic colectomy, are not currently recommended due to incomplete penetrance and substantial variation in the risk imparted by different genes and variants^27,55,61^.

#### Anti-cancer therapy

Although dMMR CRCs are associated with a lower risk of lymph node metastases, adjuvant oncological therapy is recommended when such spread occurs, yet may be less responsive to 5-fluorouracil-based chemotherapy^62,63^. Immunotherapy for metastatic CRC with dMMR or MSI using CPI targeting CTLA4 and the PD1/PD-L1 axis results in greater complete and partial clinical response rates, with long-term disease control^52,64^. CPI therapy is now considered as the first-line management of metastatic or unresectable CRC with dMMR/MSI^65^. Meanwhile, in 2022, studies of CPI in non-metastatic and resectable CRC were published, with very promising results that may represent a paradigm shift in practice for LS-associated CRC^66^. In a single-institution phase II trial of 16 participants with dMMR rectal cancer, patients were assigned to the CPI agent dostarlimab for 6 months before chemoradiotherapy and surgery. The first 12 patients on the protocol, with many cases of locally advanced tumours, all exhibited a complete clinical response as measured by clinical imaging, rectal examination, and endoscopy, resulting in a watch-and-wait approach without the need for chemoradiotherapy and surgery^66^. Similar results are reported for dMMR colon cancer^67^, with 95 per cent of the patients treated with CPI before surgery exhibiting a complete or near-complete pathological response^68^.

#### Surveillance

The standard of care for individuals with pathogenic MMR germline variants is to offer regular endoscopic surveillance by colonoscopy with polypectomy and early detection of possible incident CRC. Efficacy of colonoscopy surveillance to prevent CRC was demonstrated in a non-randomized study of individuals who opted for surveillance versus those that did not^69,70^. Mortality rate from CRC was reduced in those undergoing colonoscopy surveillance, with CRC incidence reduced by 65 per cent in those undergoing surveillance colonoscopies every 3–5 years. Importantly, selection and lead-time bias exist given the non-randomized design.

Colonoscopy surveillance should be initiated at 25 years of age for carriers of *MLH1* and *MSH2* and at 35 years of age for *MSH6* and *PMS2*^55^. The optimal interval of colonoscopies is debated, with recommendations of between 1-yearly and 3-yearly colonoscopies, given that differences in stage, survival, or CRC incidence have not been demonstrated between 1-, 2- and 3-yearly colonoscopy regimens, or the interval since the previous colonoscopy before incident CRC^33,71,72^. LS patients with previous CRCs are assumed to have additional modifiers of cancer risk^56^, and should undergo shorter postoperative intervals between surveillance colonoscopies. In a recent cost-effectiveness study, strategies with a 3-yearly colonoscopy interval were favoured to gain most life-years saved^73^.

Recent epidemiological studies have shown that the incidence of CRC remains high even under colonoscopy surveillance^34^, but the overall survival after prospectively observed incident CRCs is good, close to 90 per cent after 10 years^25,33,74^.

#### Chemoprevention

In a randomized placebo-controlled study ‘CaPP2’, enteric coated acetylsalicylic acid (ASA) of 600 mg per day for 2–4 years was shown to reduce the incidence of CRC in LS carriers by half, reflecting the trends in general population-based studies on ASA use^75,76^. There was no statistically significant increase in adverse effects with ASA although the study population was primarily middle-aged or younger. The preventive effect of ASA on CRC was observed 4 years after the therapy, and the incidence reduction was maintained for 10–20 years in follow-up. The number needed to treat with 2 years of ASA to prevent one CRC in LS was 25^77^.

### Familial adenomatous polyposis

FAP is the second most common germline predisposition to CRC with a distinct polyposis phenotype in classical presentation. It was first described in the literature in 1859, long before the role of the adenomatous polyposis coli gene (*APC*) was identified in 1991.

#### Epidemiology

The prevalence of FAP is estimated to be between 1 : 8000 and 1 : 18 000 in the general population, accounting for approximately 1 per cent of all CRCs^78,79^. In the presence of a multiple adenoma phenotype, the probability of *APC* pathogenic variants is associated with the number of adenomas^80^.

#### Penetrance

Classical FAP is a condition of full penetrance in *APC* pathogenic variant carriers and a gross polyposis phenotype with a 100 per cent risk of CRC, in almost all cases by 40 years of age^81^. The genomic position of the pathogenic germline variant results in variable penetrance, with an attenuated phenotype (aFAP) caused by variants located in codons less than 157 or greater than 1595, or alternatively spliced ninth exon. Intermediate phenotypes are caused by variants in codons 157–1249 and 1464–1595, excluding alternatively spliced ninth exon. Classical severe phenotypes are associated with variants in codons 1250–1464 and large deletions in the *APC* gene^82^. Milder phenotypes attributed to aFAP have a CRC risk of 70 per cent by 80 years of age, with the mean age of CRC onset at approximately 30 years^83^.

#### Carcinogenesis

The *APC* gene is located on chromosome 5q21, and has been termed the ‘gatekeeper’ tumour suppressor gene for CRC. Somatic *APC* mutations are frequently observed in sporadic CRC. Neoplasia in FAP follows the traditional adenoma-carcinoma sequence and so FAP has served as a model for conventional colorectal carcinogenesis. In FAP, neoplasia is initiated when the germline *APC* variant develops a second hit or LOH. The *APC* mutation disturbs the *Wnt* signalling pathway by leading to the cytoplasmic accumulation of β-catenin due to reduced degradation in crypt cells. *APC* also promotes defects in chromosome segregation fidelity, leading to increased aneuploidy and LOH in other genes^84^.

#### Clinical presentation

Polyposis is defined as greater than 10 cumulative adenomas detected in the colon or rectum, a definition that is sensitive but not specific to FAP. In classical FAP, the number of adenomatous polyps is substantially higher with hundreds or even thousands of adenomas present at a young age, while in aFAP less than 100 adenomas with later onset of CRC is observed. Additional distinction can also be made based on the family history. Approximately 30 per cent of those presenting with FAP have no family history of polyposis. Inheritance of *APC* pathogenic variants is, however, autosomal dominant. Additionally, desmoid tumours are a major source of morbidity rate and mortality rate in FAP after CRC, and cluster within families with variants in codon 1400 or to a lesser extent in codons 401–1400.

#### Identification

A personal or familial polyposis phenotype should instigate genetic testing in the proband and FDRs. As polyposis is a heterogenous clinical condition, multigene panel germline NGS including at least *APC*, *MUTYH*, *BMPR1A*, *EPCAM*, *MLH1*, *MSH2*, *MSH6*, *PMS2*, *POLD1*, *POLE*, *PTEN*, *SMAD4*, *STK11*, and *TP53* should be applied. Differential diagnoses also include more infrequent causes of polyposis, and a wider panel of genes associated with rare polyposis may be applied with *AXIN2*, *GREM1*, *MLH3*, *MSH3*, *MBD4*, *NTHL1*, *RNF43*, and *RPS20* included.

#### Surgery

Timing of prophylactic surgery to prevent CRC is the most relevant question in FAP. Symptoms and CRC are absolute indications for surgery. Colectomy in asymptomatic FAP carriers is indicated when the adenoma burden is no longer manageable by endoscopy or increases rapidly, when large polyps of >10 mm become frequent, or when high-grade dysplasia is identified^85^. It is usually appropriate to postpone surgery until the patients are physically and emotionally mature enough^86,87^.

The main options for surgery are restorative total proctocolectomy with ileal pouch-anal anastomosis (PC + IPAA) and total abdominal colectomy with ileorectal anastomosis (TC + IRA). Patients with less than 20 polyps in the rectum and less than 500 polyps in the colon are candidates for TC + IRA. However, patients with a PV between *APC* codons 1309 and 1328 are considered at high risk of more severe polyposis, and are usually considered for PC. Desmoid risk has implications for the timing of surgery as patients at high risk due to a pathogenic variant genomic position may benefit from postponing surgery to a later age. In aFAP phenotypes, the penetrance is less pronounced, and endoscopic management may be effective until later in life, for example 30–40 years of age.

#### Surveillance

Yearly colonoscopies are recommended from 15 years of age or from the time of diagnosis (if later) until surgery, after which the surveillance intervals may be relaxed depending on the phenotype and type of resection. If TC + IRA has been performed, the surveillance of the rectum is continued yearly.

#### Chemoprevention

Several chemoprevention agents have been tested in FAP to reduce the adenoma burden, yet none has provided a sustained response and shown tolerability over a long interval of time. In a placebo-controlled trial, 6 months of celecoxib therapy reduced the number of polyps by 28 per cent compared with placebo of 4.5 per cent at 12 months^88^. Rofecoxib, sulindac, difluoromethylornithine, and ASA have also been studied in randomized (placebo) controlled trials, without clear efficacy in preventing disease progression requiring surgical intervention^89^.

### Other polyposis syndromes

**Fig. 2:**
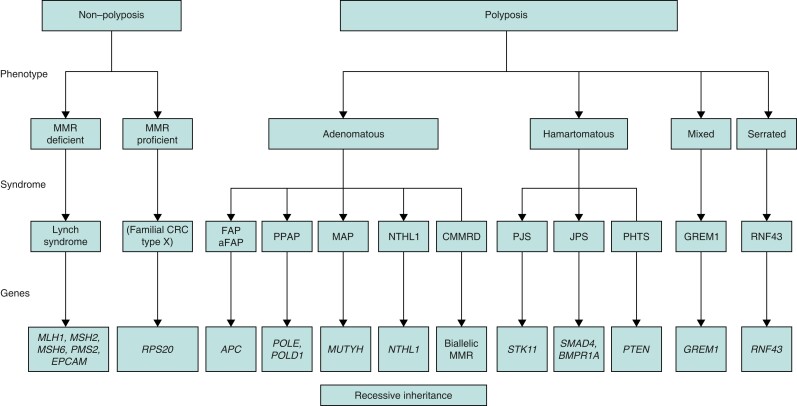
Classification of CRC syndromes MMR, mismatch repair; CRC, colorectal cancer; FAP, familial adenomatous polyposis; aFAP, attenuated familial adenomatous polyposis; PPAP, *POLE*/*POLD1*- associated polyposis; MAP, *MUTYH*-associated polyposis; CMMRD, constitutional mismatch repair deficiency; PJS, Peutz–Jeghers syndrome; JPS, juvenile polyposis syndrome; PHTS, *PTEN* hamartoma tumour syndrome. Modified from Daca Alvarez *et al*.^13^ and Valle *et al*.^90^

Numerous rare polyposis syndromes have been identified with variant confirmation required to guide management. Rarer polyposis syndromes may present with adenomatous, hamartomatous, serrated, and mixed polyposes depending on the genes involved. A simplified schematic classification of CRC predisposing germline disorders is outlined in *Fig. 2* according to Daca Alvarez *et al*.^13^ and Valle *et al*.^90^.

#### MUTYH-associated polyposis

*MUTYH*-associated polyposis (MAP) is a recessively inherited disorder associated with less than 1 per cent of CRCs^91^. *MUTYH* is a base excision repair gene on chromosome 1, and, when both alleles are affected, this leads to G → T transversions in somatic genes relevant to CRC carcinogenesis^92^. CRC lifetime incidence of 80–90 per cent without surveillance and 48 per cent under surveillance for the biallelic *MUTYH* germline defect have been reported^93^. Increased risk of CRC in monoallelic germline carriers of *MUTYH* is debated. The clinical presentation resembles that of aFAP with less than 100 polyps but also includes serrated and hyperplastic lesions primarily in the right hemicolon. Clinical guidance for MAP recommends initiating colonoscopy surveillance at 18–20 years of age with examinations 1–3-yearly and polypectomy of lesions larger than 5 mm^94^. Due to low polyp burden and rare rectal involvement, abdominal colectomy or subtotal colectomy with ileorectal anastomosis is likely sufficient to prevent metachronous CRC^95^.

#### DNA polymerase ɛ/DNA polymerase δ

Polyposis arising from germline variants of DNA polymerase ɛ (*POLE*) or DNA polymerase δ (*POLD1*) is a further autosomal dominant polyposis syndrome. Adenomas are present (10–100s) and are phenotypically dMMR/MSI with an incidence of CRC of between 32 and 63 per cent. Being hypermutated, cancers developing through this pathway may be responsive to CPI therapy^95^. Prophylactic surgery with abdominal subtotal colectomy may be appropriate with a severe polyp phenotype^96^.

#### Hamartomatous and juvenile polyposis

Hamartomatous polyposis syndromes have an estimated prevalence of 1 : 100 000–200 000. The polyps are by definition non-neoplastic with an abnormal cellular architecture and a mixture of cell types involved^97^. Syndromes presenting with hamartomatous polyps include Peutz–Jeghers syndrome (PJS), *PTEN* hamartoma tumour syndrome (PHTS), juvenile polyposis syndrome (JPS) and hereditary mixed polyposis syndrome (HMPS) (*Fig. 2*). Some polyps have distinct histomorphological features that indicate genetic germline evaluation for hereditary condition together with clinical criteria.

PJS polyps have branching bands of smooth muscle covered by hyperplastic glandular mucosa that may be present in the stomach, small bowel, and colorectum. The presence of two or more histologically confirmed PJ polyps, or any number of PJ polyps with a family history of PJS, warrant genetic testing for *STK11*. PJS has an autosomal dominant inheritance pattern with a cumulative CRC risk of 39 per cent, but also increased risk of gastric, pancreatic, breast, and other cancers^98^.

Juvenile polyps seen in JPS are most often found in the colorectum, and have dilated mucus-filling glands, prominent lamina propria, and Paneth cells, with strong immune cell infiltration. JPS is most often due to *SMAD4* and *BMPR1A* germline pathogenic variants, yet in approximately half of patients meeting clinical diagnostic criteria no causative genetic defect is found. Genetic evaluation is warranted in patients with five or more juvenile polyps in the colorectum or any number of juvenile polyps where there is also a family history of JPS^97^. Cumulative lifetime risk of CRC of 39 per cent has been quoted with a mean age of onset of 44 years^99^.

Unlike in most adenomatous polyposis syndromes, prophylactic bowel resections are usually not necessary to prevent cancer in hamartomatous and juvenile polyposis.

### Colorectal cancer summary

Relevant inherited CRC syndromes with summarized surveillance and management guidelines are presented in *Table 1*.

**Table 1:** Summary of surveillance and management guidelines in hereditary colorectal cancer syndromes

Syndrome	Lynch syndrome				FAP/aFAP	MAP	PPAP	PJS	PHTS	JPS
Genes	*MLH1*	*MSH2/EPCAM*	*MSH6*	*PMS2*	*APC*	*MUTYH* (biallelic)	*POLE/POLD*	*STK11*	*PTEN*	*SMAD4*, *BMPR1A*
Phenotype	Non-polyposis colorectal cancer				Adenomatous polyposis			Hamartomatous polyposis		Juvenile polyposis
Surveillance starting age in years	20–25*	20–25*	30–35*	30–35*	12–15	18–20	14	18 (8 for baseline)	35	12–15
Colonoscopy interval in years (healthy carriers)	2–3	2–3	2–3	3–5	1–3	1–3	2	1–3	5	1–3
Colorectal surgery implications	Subtotal abdominal colectomy advised for cancer	Subtotal abdominal colectomy advised for cancer	Standard resections for cancer	Standard resections for cancer	Prophylactic proctocolectomy or colectomy	Prophylactic colectomy	Prophylactic subtotal colectomy in severe phenotypes	Subtotal colectomy sometimes necessary for polyp burden or complications	–	Subtotal colectomy sometimes necessary for polyp burden or complications
References	^7,55^				^7,94,95,100^	^7,94,95,100^	^96^	^101,102^	^103^	^104^

FAP, familial adenomatous polyposis; aFAP, attenuated familial adenomatous polyposis; MAP, *MUTYH*-associated polyposis; PPAP, *POLE*/*POLD1*-associated polyposis; PJS, Peutz–Jeghers syndrome; PHTS, *PTEN* hamartoma tumour syndrome; JPS, juvenile polyposis syndrome. *Or 2–5 years before the earliest age of CRC diagnosis in the family (whichever is earlier).

## Hereditary gastric cancer

### Epidemiology

Gastric cancer is one of the most common cancers worldwide and is the third most common cause of cancer-related death^105^. Gastric cancer is classically divided between intestinal, diffuse, and indeterminate subtypes based on the Lauren classification, with the majority being intestinal and up to 20–30 per cent of gastric cancers classified as the diffuse subtype^106^. There are substantial region-specific differences in gastric cancer incidence, with East Asia, Eastern Europe, Central America, and South America all having increased gastric cancer incidence compared with other parts of the world^107^. Much of these region-specific differences in incidence likely result from lifestyle and environmental factors, such as *Helicobacter pylori* infection, which is well known to be associated with increased gastric cancer risk^108^.

While the majority of gastric cancers are considered sporadic, up to 20 per cent of people diagnosed with gastric cancer have a family history of gastric cancer^109^. Furthermore, up to 5–10 per cent of gastric cancers have a familial component or are due to a hereditary cancer predisposition syndrome^109^. Of the known hereditary gastric cancer risk syndromes, hereditary diffuse gastric cancer syndrome (HDGC), due to a PV in either the *CDH1* or *CTNNA1* gene, confers one of the highest lifetime risks of gastric cancer^110–112^. Other hereditary cancer risk syndromes associated with increased gastric cancer risk include LS, Li–Fraumeni syndrome (LFS), and multiple polyposis syndromes including FAP, gastric adenocarcinoma and proximal polyposis of the stomach (GAPPS), PJS, and JPS^113^. Furthermore, familial intestinal gastric cancer (FIGC) is a condition that encompasses individuals with a strong family history of intestinal-type gastric cancer with no identifiable germline PV^114^.

### Mechanisms of carcinogenesis

The classic mechanism for the development of intestinal-type gastric cancer is via the ‘Correa cascade’, which involves gastric inflammation either in the form of chronic gastritis or atrophic gastritis, followed by progression to intestinal metaplasia, dysplasia, and finally carcinoma^115^. Meanwhile some intestinal-type gastric cancers develop from precursor lesions including gastric adenomas. Diffuse gastric cancer (DGC) also arises independently of the Correa cascade^116^.

Pathogens play a major role in gastric carcinogenesis, for example *H. pylori*, estimated to infect at least half of the world’s population^117^. *H. pylori* causes chronic gastritis and promotes progression through the Correa cascade, and effective treatment can lead to reductions in gastric cancer risk^118,119^. Epstein–Barr virus (EBV) is also associated with gastric cancer, with approximately 9 per cent of gastric cancers being associated with EBV^120^.

### Identification

Evaluation for a potential hereditary cancer predisposition syndrome associated with increased gastric cancer risk is triggered by personal and family history as well as tumour analysis, as summarized in *Table 2*.

**Table 2:** Recommendations for genetic testing for hereditary diffuse gastric cancer

**Recommendations from the International Gastric Cancer Linkage Consortium for genetic testing for hereditary diffuse gastric cancer** ^121^	
Family criteria	Family history of at least two gastric cancers with one being DGC
	One DGC and one lobular breast cancer (at <70 years of age) in different family members
	At least two lobular breast cancers in the family younger than 50 years of age
Patient criteria	DGC and is either younger than 50 years of age, is of Māori ethnicity, or has a personal or family history of cleft lip or palate
	DGC and lobular breast cancer both diagnosed before 70 years of age
	Bilateral lobular breast cancer before 70 years of age
	Foci of gastric signet ring cells before 50 years of age
	
**National Comprehensive Cancer Network recommendations for genetic testing for a hereditary gastric cancer risk syndrome** ^122^	
Family criteria	Family history of gastric cancer in an FDR or SDR before 40 years of age
	Gastric cancer in two FDRs or SDRs with one diagnosis before 50 years of age
	Gastric cancer in three FDRs or SDRs
Patient criteria	Gastric cancer before 40 years of age
	Gastric cancer before 50 years of age with an FDR or SDR with gastric cancer
	Gastric cancer with at least two FDR/SDRs with gastric cancer
	Gastric and breast cancer with one diagnosis before 50 years of age
	Gastric cancer and a family history of breast cancer in an FDR or SDR diagnosed before 50 years of age
	
**Other criteria that should prompt consideration for genetic testing for a hereditary gastric cancer risk syndrome**	
	Abnormal MMR IHC of a gastric cancer
	Gastric polyposis, including hamartomatous polyposis and fundic gland polyposis, especially when there is dysplasia present and no significant history of chronic proton pump inhibitor use

DGC, diffuse gastric cancer; FDR, first-degree relative; SDR, second-degree relative; MMR IHC, mismatch repair immunohistochemical staining.

### Genetic testing

Given the increased ease of germline multigene panel testing (MGPT), increased access, and decreased cost^123^, MGPT should be the primary modality used for evaluation of suspected hereditary gastric cancer risk. In some circumstances, small multigene panel or even single gene testing may be appropriate, especially if there is a known familial PV in a gastric cancer risk gene.

At a minimum, MGPT focused on hereditary gastric cancer risk should include genes associated with HDGC (*CDH1* and *CTNNA1*), LS (*MLH1*, *MSH2*, *EPCAM*, *MSH6*, *PMS2*), FAP (*APC*), PJS (*STK11*), JPS (*SMAD4* and *BMPR1A*), and LFS (*TP53*). However, other genes may be added to this list depending on personal and family history or patient preference.

### Gastric cancer surveillance and risk management

Gastric cancer surveillance recommendations for hereditary syndromes with increased gastric cancer risk are presented in *Table 3*.

**Table 3:** Summary of recommendations for hereditary syndromes with increased gastric cancer risk

Syndrome	HDGC	Lynch syndrome	FAP/aFAP	GAPPS	PJS	JPS	LFS	FIGC
Genes	*CDH1*, *CTNNA1*	*MLH1*, *MSH2/EPCAM*, *MSH6*, *PMS2*	*APC*	*APC* promoter 1B	*STK11*	*SMAD4*, *BMPR1A*	*TP53*	Unknown
Surveillance starting age in years	18–20	30–40	20–25	15	18 (8 for baseline)	12–15	25	40–60 (or 5 years before earliest cancer diagnosis)
Upper endoscopy interval (healthy carriers)	1 year (until prophylactic gastrectomy peformed)	2–4 years	3 months–5 years	1 year (until prophylactic gastrectomy peformed)	2–3 years	1–3 years	2–5 years	1–3 years
Surveillance considerations	Modified Cambridge protocol or Bethesda protocol should be used Inlet patches should be documented and biopsied	Biopsies of the gastric antrum and body should be performed to assess for *H. pylori*, gastric intestinal metaplasia, and autoimmune gastritis	Surveillance interval should be based on the Spigelman score and/or gastric pathology A baseline upper endoscopy should be performed before 20 years of age if earlier colectomy is planned	–	–	For a clinical diagnosis of JPS without an *SMAD4* or *BMPR1A* PV, the surveillance interval can be increased to 5 years in the absence of gastric polyps	–	–
Surgical considerations	Prophylactic total gastrectomy between 20 and 30 years of age	–	If total gastrectomy is performed, Roux limb should be constructed to allow for continued duodenal surveillance	Prophylactic total gastrectomy by the 30s	–	–	–	–
References	^121,124^	^125^	^126^	^126^	^97,126^	^97,126^	^127^	^114,128^

–, no clear guidance established for the condition; HDGC, hereditary diffuse gastric cancer syndrome; FAP, familial adenomatous polyposis; aFAP, attenuated familial adenomatous polyposis; GAPPS, gastric adenocarcinoma and proximal polyposis of the stomach; PJS, Peutz–Jeghers syndrome; JPS, juvenile polyposis syndrome; LFS, Li–Fraumeni syndrome; FIGC, familial intestinal gastric cancer.

#### Hereditary diffuse gastric cancer syndrome

HDGC is associated with PVs in either the *CDH1* or *CTNNA1* gene^129–131^ and confers the highest risk of DGC amongst all of the known hereditary gastric cancer risk syndromes. For *CDH1* carriers, early estimates of DGC risk were as high as 83 per cent by 80 years of age^132^; however, more recent data, ascertained with less bias toward gastric cancer predominant families, have shown gastric cancer risks of 33–42 per cent by 80 years of age^110,111^. The first risk estimate in *CTNNA1* carriers was also recently reported showing a DGC risk by 80 years of age of 49–57 per cent^112^, yet only included families with DGC. Therefore, the quoted lifetime gastric cancer risk in *CTNNA1* carriers will also likely decrease as more families are identified. Indeed, a recent report of *CTNNA1* phenotypes on MGPT showed that only 12 per cent of patients and 21 per cent of families with a *CTNNA1* loss-of-function variant had a history of gastric cancer^133^.

The major gastric cancer risk management decision for carriers of a PV in *CDH1* or *CTNNA1* is whether to pursue endoscopic surveillance or risk-reducing total gastrectomy. Current gastric surveillance recommendations from the International Gastric Cancer Linkage Consortium (IGCLC) for carriers of a PV in *CDH1* or *CTNNA1* help inform this decision and are based on family history of DGC^121^. If there is a family history of DGC along with a PV in *CDH1* or *CTNNA1*, then risk-reducing total gastrectomy should be considered, with preoperative endoscopy performed to identify whether gastric cancer has already developed. If there is no family history of gastric cancer, carriers of a PV in *CDH1* or *CTNNA1* may consider either risk-reducing total gastrectomy or yearly surveillance.

Current recommendations for endoscopic surveillance support the use of a modified Cambridge biopsy protocol, which includes targeted biopsies of any macroscopic gastric lesions, 28–30 non-targeted biopsies of the antrum (5), transition zone (5), body (10), fundus (5), and cardia (3–5), and documentation and biopsy of any oesophageal inlet patches^121^. It is important that these biopsies are reviewed by a specialized gastrointestinal pathologist to facilitate identification of foci of signet ring cell carcinoma (SRCC), which indicate the need for risk-reducing total gastrectomy. However, despite extensive biopsy sampling, foci of SRCC are still frequently observed on subsequent gastrectomy specimens, even in the setting of negative biopsies^134^. The Bethesda protocol^124^ proposes a total of 88 non-targeted biopsies, with evidence of increased detection of foci of SRCC, yet a false negative rate of 38 per cent. Advanced endoscopic methods including endoscopic ultrasound^135^ and confocal laser endomicroscopy^136^ have been studied without clear increases in SRCC detection^137^. At this time, there are no reliable strategies for chemoprevention of gastric cancer in HDGC, or therapies to selectively target and eliminate SRCC, although this is an area of active research in preclinical models^138,139^.

Given the lack of reliable methods to adequately identify or treat foci of SRCC in HDGC, risk-reducing total gastrectomy is often the preferred strategy with debate about timing. A recent modelling study recommended the optimal age for risk-reducing total gastrectomy as 39 years for men and 30 years for women^140^, whereas recommendations from the IGCLC advise risk-reducing total gastrectomy between 20 and 30 years of age^121^. There is also no role for partial gastrectomy in the management of HDGC and, where risk-reducing total gastrectomy is performed, it should be done in a centre with HDGC expertise. If foci of SRCC are identified on index endoscopy, neoadjuvant therapy is not necessary before gastrectomy in the absence of a more invasive gastric cancer. Furthermore, it is critically important to confirm both proximal and distal margins of the gastrectomy specimen to ensure that no gastric tissue remains. Recent reports have highlighted favourable safety outcomes amongst HDGC patients undergoing risk-reducing total gastrectomy^141,142^.

Individuals who have a risk-reducing total gastrectomy need long-term follow-up for both nutritional monitoring and other cancer screening. Enhanced breast cancer screening is recommended for carriers of a PV in *CDH1* or *CTNNA1*, yet current guidelines do not recommend regular CRC screening^121^ given average rates of colorectal neoplasia in *CDH1* carriers compared with the general population^143^. A recent consensus panel provided recommendations for longitudinal monitoring of *CDH1* carriers after a risk-reducing gastrectomy focusing on nutrient monitoring and supplementation as well as bone health^144^.

#### Lynch syndrome

LS due to a PV in *MLH1*, *MSH2*, *MSH6*, *PMS2*, or *EPCAM* is one of the most common gastric cancer predisposition syndromes, affecting 1 in 279 individuals^145^. Gastric cancer risk in LS can be up to 9 per cent, however, but varies by genotype, with *PMS2* variants associated with lower gastric cancer risk than other variants^125^. Male sex, having an FDR with gastric cancer, and older age are also associated with a higher risk of gastric cancer in LS^146^. Debate surrounds gastric surveillance in LS: when it should begin and how often it should be performed. The National Comprehensive Cancer Network (NCCN) has recently recommended routine gastric surveillance for *MLH1*, *MSH2*, *MSH6*, and *EPCAM* carriers beginning between 30 and 40 years of age and repeating every 2–4 years^126^, while the BSG, the European Hereditary Tumour Group (EHTG), and the European Society of Coloproctology (ESCP) do not recommend routine gastric surveillance in LS outside of clinical trials^7,55^.

Studies of gastric surveillance in LS from the USA^147–149^ and Europe^125,150^ have illustrated that surveillance leads to clinically actionable findings as well as early-stage gastric cancers. A recent meta-analysis of upper gastrointestinal surveillance demonstrated that the pooled event rate for detecting an upper gastrointestinal cancer was 0.9 per cent, a high-risk lesion was 4.2 per cent, and an actionable finding was 6.2 per cent^151^. However, the impact of upper gastrointestinal surveillance on survival in LS remains unknown.

When gastric surveillance is performed, *H. pylori*, gastric intestinal metaplasia, and autoimmune gastritis should be assessed via biopsy of the gastric antrum and body^125^. If *H. pylori* is detected, the patient should be treated, with confirmation of eradication. Even if a decision is made to forego gastric surveillance in LS, testing for *H. pylori* should still be performed non-invasively, especially if there is consideration of starting aspirin for chemoprevention. While aspirin has not been shown to have a chemopreventive effect on gastric cancer in LS^75^, recent evidence from the randomized controlled CaPP2 trial has indicated that resistant starch may decrease upper gastrointestinal cancer risk, including gastric cancer risk^152^.

Development of gastric cancer in LS during or outside of active surveillance, unlike HDGC, does not immediately dictate the need for a total gastrectomy, with limited resections appropriate depending on tumour location. Furthermore, treatment with immunotherapy can be considered for unresectable, metastatic, dMMR disease^153^.

#### Familial adenomatous polyposis/gastric adenocarcinoma and proximal polyposis of the stomach

FAP is associated with increased gastric cancer risk. This risk of gastric cancer in FAP has been reported to be as high as 7.1 per cent. While higher estimates have been seen in primarily East Asian populations^126^, there are more recent reports of an increase in gastric cancer incidence in FAP in the West as well^154^. Similar to FAP, GAPPS is an autosomal dominant condition associated with increased gastric cancer risk^155^. However, unlike FAP, which is due to PVs within the coding region of *APC*, GAPPS results from point mutations in promoter 1B of the *APC* gene, which selectively result in gastric polyposis without polyposis noted in the small and large bowel^156^. Reported estimates of gastric cancer risk in GAPPS are still evolving, but have ranged between 12 and 25 per cent^155,157^.

Individuals with FAP and GAPPS frequently have fundic gland polyposis, often with low-grade dysplasia^158^. However, it is thought that high-risk lesions such as gastric adenomas, pyloric gland adenomas, and gastric lesions with high-grade dysplasia are the most likely cancer precursor lesions, as these lesions are observed more frequently in individuals with gastric cancer^159^. Additionally, carpeting of gastric polyps, gastric polyps larger than 2 cm, and mounds of gastric polyps are other endoscopic features associated with increased gastric cancer risk in FAP^159^.

Upper gastrointestinal tract surveillance in FAP has classically been guided by the Spigelman stage of duodenal polyposis, with an interval between 3 months and 5 years depending on the duodenal polyp burden^126^. However, with recent increases in gastric cancer incidence in FAP, gastric polyp burden may be used to dictate gastroduodenal surveillance intervals. Proposed surveillance for gastric polyposis in FAP includes basing surveillance intervals on the number, size, pathology (including presence of dysplasia), and whether or not there are mounds of gastric polyps, and making this recommendation independent of duodenal findings^154^. Recommendations for when to start upper gastrointestinal surveillance in FAP vary between 20 and 25 years of age; however, if a patient with FAP is undergoing another abdominal surgery and has not yet had a baseline upper endoscopy, it is prudent to first obtain a preoperative oesophagogastroduodenoscopy to rule out upper gastrointestinal neoplasia. Data on gastric surveillance in GAPPS are much more limited, with current recommendations to start gastric surveillance at 15 years of age, and repeat surveillance yearly^126^.

Risk-reducing total gastrectomy should be offered to patients with GAPPS by approximately 30 years of age, yet earlier surgery may be considered based on patient preference, polyp burden, and family history^160^. In FAP, gastric surgery should only be considered for individuals who develop gastric cancer, or who develop high-risk gastric lesions that cannot be managed and/or surveilled endoscopically. If surgery for gastric neoplasia is performed in FAP, a total gastrectomy can be considered based on the polyp burden and given the risk of metachronous gastric neoplasia. In the setting of a total gastrectomy, special attention should be paid to construction of the Roux limb, as continued duodenal surveillance will be necessary, and may pose an endoscopic challenge after surgery.

#### Hamartomatous and juvenile polyposis syndromes

Both PJS and JPS have been associated with increased gastric cancer risk. PJS, which is diagnosed by having a PV in *STK11* or through meeting clinical diagnostic criteria, has been associated with a cumulative gastric cancer risk of 29 per cent^161^. Individuals with PJS are recommended to undergo upper endoscopic surveillance starting in childhood (age 8–10 years); however, at this age surveillance is primarily aimed at detection of polyps, which may lead to anaemia, obstruction, or intussusception^97^. In adulthood, upper gastrointestinal surveillance is recommended to occur at least every 2–3 years^97^.

JPS can result from a PV in *SMAD4* or *BMPR1A*; however, in nearly half of cases there is no PV identified and instead patients meet clinical diagnostic criteria for JPS^97^. In JPS, gastric cancer has primarily been reported in *SMAD4* carriers, with a recent study showing a gastric cancer incidence of 27 per cent in *SMAD4* PV carriers^162^. Furthermore, there appears to be a distinct genotype–phenotype correlation with gastric polyposis in JPS between those with and without an identifiable PV. In a recent multicentre study, individuals with JPS without a *SMAD4*/*BMPR1A* PV had no reported gastric cancer or any reported gastric polyps^163^. Upper gastrointestinal cancer surveillance in JPS has been recommended to begin as early as 12–15 years of age and be repeated every 1–3 years; however, consideration can be made to starting upper gastrointestinal cancer surveillance later and using longer intervals in individuals without a PV in *SMAD4*/*BMPR1A*^97,126^.

#### Other gastric cancer risk syndromes

Other hereditary syndromes associated with increased gastric cancer risk include LFS, hereditary breast and ovarian cancer (HBOC) syndrome, and FIGC. In LFS, 3.3 per cent of individuals and 5.9 per cent of LFS families were reported to have gastric cancer^164^, with higher rates reported in Asian LFS populations^165^. Some groups have recommended upper gastrointestinal surveillance as a routine part of LFS care^127^; however, this recommendation is not uniform^166^. HBOC due to a PV in *BRCA1* or *BRCA2* is well documented to increase the risk of breast, ovarian, pancreatic, and prostate cancer^167,168^. However, several recent studies have also highlighted an increased risk of gastric cancer^168,169^. Despite this increased risk, there are no recommendations for gastric surveillance in *BRCA1/2* PV carriers, although if a carrier does develop a *BRCA1/2*-related gastric cancer, these individuals may be eligible for specific therapy such as poly ADP ribose polymerase (PARP) inhibitors^170^. Finally, FIGC is a hereditary intestinal-type gastric cancer risk syndrome that at this time does not have an identifiable genetic aetiology^114^, but is instead diagnosed based on clinical criteria^128^. As this is a newly characterized syndrome with limited data, the cancer risk associated with FIGC remains poorly characterized, and at this time there are no consensus guideline recommendations about surveillance for affected individuals.

## Hereditary pancreatic cancer

### Epidemiology

Pancreatic cancer is a devastating malignancy with a rising worldwide incidence and mortality rate^171^.

In early-stage disease, diagnosis relies upon the availability of advanced cross-sectional imaging with the capacity to access tissues for diagnostic biopsy (often via endoscopic ultrasonography). Survival is strongly associated with stage at diagnosis, yet patients are often diagnosed at an advanced stage, restricting therapeutic options largely to palliative measures^172^. There are no early diagnosis tools available for broad generalized use amongst the population. When found at an early stage, cure is achieved with surgical resection with or without systemic chemotherapy.

### Mechanisms of carcinogenesis

There are two main histological pathways leading to exocrine cancer of the pancreas, or pancreatic ductal adenocarcinoma. The first involves dysplastic progression of the cells lining the branches of the exocrine collecting system, both the acinar cells and ductal cells. These dysplastic changes on histological examination are accompanied by mutations in four major driver genes commonly seen in pancreatic cancer: *KRAS*, *CDKN2A*, *TP53*, and *SMAD4*^173^. Both *KRAS* mutations and alterations in *CDKN2A*, including homozygous loss, are thought to be the earliest events in pancreatic tumorigenesis, with *SMAD4* loss thought to be a relatively late event^174^.

The second histological pathway tends to involve cystic growth of the exocrine ductal tree and accumulated dysplasia within these cysts^175^. These features are termed intraductal papillary mucinous neoplasms (IPMN). Importantly, not all IPMN progress to malignancy and the clinical management of IPMN can be controversial as the only effective strategy thus far employed to eliminate the potential risk of underlying malignancy is surgical resection. The molecular changes that accompany IPMN-associated dysplasia share some common traits to the pathway described in pancreatic intraepithelial neoplasia (PanIN), but, IPMN-associated dysplasia can also be driven by mutations outside the big four, including mutations in *GNAS*, *RNF43*, and numerous others^176^. Given the wider variety of somatic mutations found in this population, individuals with IPMN and a strong family history have been a focus of several studies investigating unique genetic drivers of disease and clinical paradigm screening for high-risk cohorts^177^. Interestingly, the underlying molecular mechanisms driving the hereditary nature of this disease in many cohorts remain uncertain.

### Clinical presentation and identification

Patients at increased risk, such as those carrying a hereditary risk, provide an opportunity to increase the detection of premalignancy or early malignancy through screening and so the ultimate long-term survival and cure of cancer in these patients is more likely than with sporadic cancer^178^. Screening in pancreatic cancer is limited by the capacity, associated costs, and potential for discovery of other incidental findings of uncertain significance, driving potential harm. Currently proposed screening paradigms rely heavily on short-interval cross-sectional imaging (with CT, MRI, and magnetic resonance cholangiopancreatography (MRCP) alongside endoscopic ultrasound evaluation of the gland. Blood-based diagnostic biomarkers, or liquid biopsy, is an area of active translational research.

Given the intensity of screening paradigms, there is a natural tension in identifying an optimal cohort meeting a threshold whereby the benefits outweigh the risks. A family history of pancreatic cancer is a significant predictor of disease risk, with some series suggesting a six-fold or higher risk compared with the general population^179^, with an increasing number of family members affected with an elevated risk^180^. The risk is also increased for kindred in whom disease onset was particularly young (that is less than 50 years of age)^180^. Taken together, the International Cancer of the Pancreas Screening Consortium recommends that screening programmes are implemented for individuals with an estimated lifetime risk of 5 per cent or greater^181^. This corresponds to individuals with a known deleterious germline variant for pancreatic cancer as well as individuals with a strong family history of pancreatic cancer. The generally accepted threshold for screening based upon family history alone is the presence of at least two FDRs with a history of pancreatic cancer, or at least one FDR and one second-degree relative with the disease who are themselves directly related^181^. Genetic testing in the presence of a strong family history is not a prerequisite to enter into screening programmes in this disease—though the two groups, familial risk only and known genetic susceptibility, are often grouped independently for analysis in the literature.

### Pancreatic cancer susceptibility gene variants

The value of identifying kindred at risk of pancreatic cancer and potentially intervening prior to cancer development or during early stages, has led to recommendations for example from the NCCN that all patients undergoing therapy for pancreatic cancer receive germline assessment^182^. When panel testing is utilized in this cohort, even in the absence of known familial risk, nearly 10 per cent of cases are identified as being linked to high-penetrance genetic variants^183,184^. Common variants identified include *BRCA1/BRCA2*, MMR genes associated with LS, *CDK2NA*, and *ATM*. Less commonly, PVs in genes such as *TP53*, *PRSS1*, *STK11*, and *PALB2* can be present, which can increase the relative risk of developing pancreatic cancer 30-fold or more. Many of these gene variants also carry the risk of developing another gastrointestinal malignancy.

The most frequently identified genetic risk factors are pathogenic variants identified in *BRCA2* and *ATM.* When screening is employed for all patients, regardless of familial risk, the prevalence for each approaches 2 per cent^184^. Founder variants are important to recognize in certain populations such as the Ashkenazi Jewish population and *BRCA2* families. In patients with these deleterious founder variants, the lifetime risk of pancreatic cancer development approaches 10 per cent by 80 years of age. The lifetime risk conveyed by *BRCA1* in pancreatic cancer is significantly lower (estimated to be 3 per cent) and falls below the general cut-off risk to initiate screening. *ATM* variants are associated with a clinical diagnosis of ataxia telangiectasia and, similar to *BRCA2*, convey an approximate lifetime risk of pancreatic cancer approaching 10 per cent^185^.

The lifetime risk of pancreatic cancer is highest for patients with known germline variants in three unique genes: *STK11*, *CDK2NA*, and *PRSS1*. In addition to the inherited risk of CRC and gastric cancer, PVs in *STK11* and a diagnosis of PJS are associated with a lifetime risk of pancreatic cancer that exceeds 30 per cent in some series^186^. Another founder variant is responsible for many of the cases seen in *CDKN2A* families (the Dutch founder variant affecting the p16 isoform of the protein). In these cases, the inherited predisposition can be elucidated with a strong familial history of melanoma likely due to impaired cell-cycle regulation. This syndrome, known as familial atypical mole and multiple melanoma syndrome, is associated with a lifetime risk of pancreatic cancer approaching 20 per cent^187^. Finally, PVs in the *PRSS1* gene are most often found when evaluating patients with both a personal and a family history of recurrent acute pancreatitis. The *PRSS1* protein encodes a pancreatic trypsinogen that, when mutated, can lead to inappropriate trypsin activation within the pancreatic parenchyma. The resulting clinical syndrome can result in the development of malignancy in up to 40 per cent of patients in some studies^188^. The risk of malignancy in this pancreatitis cohort is high enough that multidisciplinary programmes are beginning the process of creating shared decision-making models that propose prophylactic total pancreatectomy in highly selected individuals^189^.

Other less frequently encountered hereditary variants impacting pancreatic cancer risk are found in the MMR genes causing LS (4 per cent lifetime risk), *PALB2* (2 per cent lifetime risk), *TP53* causing LFS (1–2 per cent lifetime risk), *CPA1* and *CPB1* (0.5 per cent lifetime risk), and other hereditary pancreatitis genes such as *CFTR* and *SPINK-1* (5–20 per cent lifetime risk)^178,186,190^.

### Pancreatic cancer surveillance and risk management

Once the decision to undertake routine surveillance for patients at high risk has been made, the optimal screening strategy for this disease should include routine interval history and physical exams supplemented by multimodal imaging assessment and, for a select few, laboratory profiling. The optimal strategy is hotly debated in the literature and is perhaps most easily reconciled by focusing first on the underlying patient features associated with each hereditary risk case. One management strategy to manage patients with a hereditary risk, and clinical manifestations resulting in pancreatic cystic disease, is to adapt the Fukuoka guidelines^191^. The strategy for patients with hereditary risk and clinical manifestations of pancreatitis has been outlined by an international consensus committee from the International Association of Pancreatology, the American Pancreatic Association, the Japan Pancreas Society, and the European Pancreatic Club^192^. Lastly, and most controversially, the most common screening guidelines used for patients without pancreatic cystic disease or pancreatitis tend to follow along the protocols detailed by the International Cancer of the Pancreas Screening Consortium^181^.

For patients with pancreatic cystic disease, the risk of developing pancreatic malignancy is assessed by screening for a set of clinical, radiographic, and laboratory criteria defined as ‘high-risk stigmata’ and/or ‘worrisome features.’ These features are identified by a strategy that employs both cross-sectional imaging and endoscopic ultrasound assessment. Importantly, the Fukuoka guidelines do not take hereditary risk or family history into account and, therefore, should be adapted with some recognition of underlying risk. The decision to resect these lesions, particularly in the absence of a tissue diagnosis suggesting high-grade dysplasia or malignancy, relies on shared decision-making to outline the risks for malignancy suggested by these guidelines against the risks associated with a surgical procedure to extirpate the associated cystic disease. Surgical procedures involve formal gland resection with either Whipple’s procedure or a distal pancreatectomy as limited enucleations result in morbid pancreatic leak and are of limited oncological benefit. In the absence of surgical resection, surveillance strategies are suggested by the Fukuoka guidelines in a manner that is tailored to underlying risk^191^. Notably, this commonly utilized strategy to assess patients with cystic disease does not take underlying hereditary risk into account and conversations during shared decision-making often hinge on estimating this somewhat nebulous added risk. Finally, a comparison of this method for risk identification in patients with cystic disease with the guidelines outlined by the International Cancer of the Pancreas Screening Consortium (that take the presence of cystic disease into limited account) found both methods to be moderately specific but with limited sensitivity, suggesting more work focused in this space may lead to improved outcomes^193^.

In patients with pancreatitis expert agreement recommends screening in patients with *PRSS1* PV but no routine screening for patients with *SPINK1* and other PVs^192^. Screening in this cohort was recommended to be initiated at 40 years of age; however, the ideal screening modality and interval remains controversial. Both CT and MRI were mentioned as reasonable strategies for screening, with preference deferred to locoregional high-volume expert centres. Endoscopic ultrasound, while used in the assessment for cyst patients, was not recommended in the setting of pancreatitis (weak consensus agreement) due to fibrosis, inflammation, and calcification, which can impair assessment. Recommendations to avoid tobacco, limit alcohol, and maintain a healthy diet with integration of physical exercise were strongly agreed to; other meaningful risk mitigation strategies were scarce, reflective of the underlying evidence available for interpretation.

In patients without cystic disease or pancreatitis, pancreatologists rely upon guidelines detailed by the International Cancer of the Pancreas Screening Consortium^181^. The programme of the International Cancer of the Pancreas Screening Consortium is grounded in a Delphi approach with expert consensus leading to 55 statements of agreement reflective of modern practice. The core of the recommendations included a firm statement on those eligible for enhanced screening programmes (discussed above), the age to begin screening in high-risk individuals, the modality and timing used for screening, and the indications to consider surgical resection. In this consortium, experts recommend beginning screening at 50 years of age or 10 years younger than the age at which the first relative was affected, whichever is sooner. Both MRI/MRCP and endoscopic ultrasound are noted to be effective screening modalities in this cohort, though preference for one over another, strategies to incorporate both, and indications to integrate CT into screening programmes did not reach consensus. Recent data from the Cancer of Pancreas Screening-5 study demonstrated that screening of high-risk individuals led to downstaging of detected pancreatic cancers and dramatically improved survival compared with pancreatic cancers detected outside of screening^194^. Finally, there were no indications identified for prophylactic pancreatectomy in the absence of identified lesions during screening as the main goals remain to identify high-grade dysplastic precursor lesions and T1N0M0 cancer.

In pancreatic cancer, hereditary predisposition can be assigned in approximately 10 per cent of patients. Screening in both the general population and high-risk individuals remains limited by the occult nature of the disease, a lack of diagnostic biomarkers, and the low sensitivity of high-quality imaging for disease onset. The risk of disease is balanced against the risks involved for treatment, namely radical oncological resection, in modern guidelines for management of high-risk individuals.

## Data Availability

Not applicable.

## Summary

Hereditary gastrointestinal cancers are under-diagnosed and require attention for identification and differential diagnostics with easy access to germline genetic evaluation to confirm the diagnosis. Awareness of multiple predisposing conditions to both gastrointestinal and extra-intestinal cancers is imperative in the identification of specific germline syndromes and resulting surveillance and management according to relevant condition-specific clinical guidelines.
